# Advances in precision therapy of low-grade serous ovarian cancer: A review

**DOI:** 10.1097/MD.0000000000034306

**Published:** 2024-04-26

**Authors:** Qing Wang, Sheng-Han Cao, Yan-Yu Li, Jing-Bo Zhang, Xin-Hui Yang, Bei Zhang

**Affiliations:** a Department of Obstetrics and Gynecology, Xuzhou Central Hospital, Xuzhou, Jiangsu, China; b Graduate School of Bengbu Medical University, Bengbu, Anhui, China.

**Keywords:** hormone therapies, LGSOC, molecular characteristics, surgical, targeted therapies

## Abstract

Low-grade serous ovarian carcinoma (LGSOC) is a rare subtype of ovarian cancer that accounts for approximately 6% to 10% of serous ovarian cancers. The clinical treatment of LGSOC is similar to that of high-grade serous ovarian carcinoma, however, its clinical and molecular characteristics are different from those of high-grade serous ovarian carcinoma. This article reviews the research on gene diagnosis, surgical treatment, chemotherapy, and biological therapy of LGSOC, providing reference for clinical diagnosis and treatment of LGSOC. Surgery is the cornerstone of LGSOC treatment and maximum effort must be made to achieve R0 removal. Although LGSOC is not sensitive to chemotherapy, postoperative platinum-based combination chemotherapy remains the first-line treatment option for LGSOC. Additional clinical trials are needed to confirm the clinical benefits of chemotherapy and explore new chemotherapy protocols. Hormone and targeted therapies may also play important roles. Some patients, particularly those with residual lesions after treatment, may benefit from hormone maintenance therapy after chemotherapy. Targeted therapies, such as MEKi, show good application prospects and are expected to change the treatment pattern of LGSOC. Continuing to further study the genomics of LGSOC, identify its specific gene changes, and combine traditional treatment methods with precision targeted therapy based on second-generation sequencing may be the direction for LGSOC to overcome the treatment bottleneck. In future clinical work, comprehensive genetic testing should be carried out for LGSOC patients to accumulate data for future scientific research, in order to find more effective methods and drugs for the treatment of LGSOC.

## 1. Introduction

Epithelial ovarian cancer (EOC) accounts for approximately 90% of ovarian malignancies and serous ovarian cancer accounts for approximately 75% of EOC cases. In 2014, the World Health Organization (WHO) classified EOC into types I and type II.^[1,2]^ EOC type I is a low-grade malignant tumor, belonging to the genetically stable type, and the main pathological types are low-grade serous ovarian carcinoma (LGSOC), mucinous carcinoma, endometrioid carcinoma, and clear cell carcinoma. Type II EOC is a highly malignant tumor that includes high-grade serous ovarian carcinoma (HGSOC), undifferentiated carcinoma, and malignant meso-blastocyst mixed tumors. It has a high degree of genetic instability and is characterized by TP53 mutations.^[3]^ LGSOC and HGSOC are both serous cancers; however, they have their own clinical manifestations, pathological features, pathogenesis, and molecular biological characteristics. LGSOC is usually secondary to serous borderline tumors (SBOT), and changes in the KRAS-BRAF-MEK-MAPK signaling pathway play an important role in its occurrence and development, often accompanied by KRAS, BRAF, or NRAS mutations. LGSOC is more common in young women, with a long clinical course and a good prognosis, but prone to chemotherapy resistance.^[1,4]^ HGSOC mostly comes from the umbrellum epithelium of the fallopian tube, and is often accompanied by TP53 or BRCA mutations. No prodrome lesions can be found in the ovary itself, presenting a highly invasive course and a poor prognosis.^[5]^ Most key clinical trials on clinical interventions for ovarian cancer are based on HGSOC studies. However, with increasing research on the clinicopathology and molecular genetics of LGSOC, the conclusions of HGSOC studies can no longer be unconditionally applied to LGSOC. At present, a large amount of research data have identified the molecular pathology and major gene regulatory pathways of LGSOC.^[6]^ This paper will review the molecular characteristics, clinical diagnosis, treatment difficulties of LGSOC, new treatment strategies to promote the update of the diagnosis and treatment concept of LGSOC, and provide a reference value for the basic research and clinical treatment of LGSOC.

## 2. Tissue origin and diagnosis of LGSOC

Ovarian cancer is the most lethal gynecological malignancy.^[7]^ The high fatality rate associated with ovarian cancer is not only due to the challenges in early stage diagnosis and development of drug resistance, but also due to the uncertainty regarding cancer origin and pathogenesis.^[8]^ The tissue origin of ovarian cancer has successively come from the theory of “ovarian surface epithelia (OSE)” and the theory of “second mullerian system” outside the ovary. However, the real evidence that ovarian HGSOC originates from outside the ovary starts from the tissue samples of patients with hereditary BRCA gene mutations who have undergone preventive resection of the fallopian tubes and ovaries. The intraepithelial lesions of fallopian tubes similar to ovarian HGSC cells are found in the epithelium of fallopian tubes and are now called serous tubal intraepithelial carcinoma, with an occurrence probability of 38%, while no serous carcinoma was found in the corresponding ovarian tissue.^[9]^ Molecular studies have also suggested that ovarian HGSOC and its matched serous tubal intraepithelial carcinoma have the same gene changes, including changes in the gene copy number. Genetic analysis of the oviduct epithelium, ovarian surface epithelium, and corresponding tumor tissues of ovarian HGSOC patients showed that the gene expression pattern of ovarian HGSOC was more similar to that of the oviduct epithelium.^[10]^ Compared with HGSOC, the origin of LGSOC is still unknown, and the limited number of LGSOC cases is the main obstacle to this study. There is a hypothesis that the pathogenesis mode of LGSOC is: OSE→OEI (ovarian epithelial inclusion)→serous cystadenoma→borderline serous cystadenoma→low-grade serous carcinoma. Additionally, the correlation between the histomorphology and molecular pathway of LGSOC and SBOT is more than 75%, which strongly supports this hypothesis.^[11]^ Nevertheless, Kurman et al^[12,13]^ observed that the cell composition and immunophenotype of ovarian epithelial inclusions are the same as those of oviduct mucosa epithelium and are completely different from the ovarian surface germinal epithelium. They proposed that low-grade serous carcinoma is likely to originate from the oviduct mucosa epithelium, that is, the origin theory of papillary tubal hyperplasia (PTH) of the oviduct epithelium. It is believed that the primary site of LGSOC did not originate from the ovary, and the evidence was that 91% of LGSOC patients had papillary proliferative lesions in the oviduct epithelium; therefore, it was inferred that PTH was a precancerous lesion of serous tumors, while Kurman et al induced the proliferation of oviduct mucosa epithelium through chronic inflammation, and subsequently developed into PTH. They successfully constructed a low-grade pelvic serous hyperplasia model, including ovaries, extraovarian SBOT, noninvasive planting lesions, and oviduct endometriosis. According to this hypothesis, the epithelium of the fallopian tube falls off and is planted on the surface of the ovary and peritoneum, resulting in secondary endometriosis of the fallopian tube. On this basis, atypical hyperplasia occurs, which subsequently develops into SBOT and LGSOC. Studies by Qiu et al^[8]^ and others conducted an RNA-seq analysis on 31 tissue samples of LGSOC, serous borderline tumor, oviduct epithelial cells, OSE cells, human peritoneal mesothelial cells, and HGSOC (control group). The results showed that the gene expression of LGSOC and oviduct epithelial cells was highly consistent compared with that of other cells, which further verified the hypothesis of oviduct origin at the molecular level.

## 3. Molecular change characteristics of LGSOC

The interest in molecular studies of cancer management has increased substantially in the recent years. Exploring the molecular profile of a specific cancer should enhance the understanding of its clinical behavior and aid in developing an active targeted therapy.^[14]^ Increasing evidence shows that the mitogen-activated protein kinase (MAPK) pathway is an important molecular pathway of LGSOC.^[15–18]^ MAPK is an important protein kinase system in cells, which mediates the transmission of extracellular signals from the cell membrane to the nucleus, and is an important molecular pathway for tumorigenesis and development.^[17,18]^ More than half of LGSOC continuously activate the MAPK pathway, and their characteristic mutant genes include KRAS, BRAF, and NRAS.^[19]^ Several studies^[15,16,19]^ have evaluated the mutation of LGSOC-related genes and found the KRAS mutation rate (15.8%–54.4%), BRAF mutation rate (2%–32%), NRAS mutation rate (3.6%–22%), and there was a mutual exclusion of mutations. Hunter et al^[16]^ found that the mutation rate of KRAS/BRAF/NRAS is 83% in SBOT and 63% in LGSOC. Contrastingly, the mutation frequency of KRAS in SBOT and LGSOC was similar, whereas BRAF mutations were more common in SBOT. Scholars have speculated that the BRAF gene mutation only participates in the formation of SBOT, but decreases the ability of the tumor to continue to progress. Therefore, the relative incidence of BRAF gene mutations in LGSOC is low,^[20,21]^ and patients with BRAF mutations are associated with early disease and good prognoses.^[20]^ Emmanuel et al^[22]^ found that NRAS mutations are unique to LGSOC and do not exist in SBOT. It was inferred that NRAS may be a carcinogenic driving gene of LGSOC. In order to study the relationship between gene changes and clinical outcomes, and determine the prevalence of pathogenic germline mutations in LGSOC, Manning-Geist et al^[23]^ sequenced 119 LGSOC patients based on a panel (505 genes), collected somatic cell and germline mutations, and related clinical characteristic data, and the results were announced at the International Gynecological Oncology Society Global Annual Meeting in 2021, 60% of LGSOC found changes in the MAPK pathway (KRAS, NRAS, BRAF, and EIF1AX), 8.9% of the patients carried pathogenic germline mutations; however, none of the patients had BRCA 1/2 mutations. The changes of the MAPK pathway and platinum sensitivity are significantly related to the prolongation of patient survival, while the changes of MAPK pathway and prolongation of patient survival are not related to platinum sensitivity, which suggests that the mutation of this pathway is related to the improvement of tumor outcomes. The latest study on LGSOC genomics not only confirmed the dominant position of KRAS-BRAF-MEK-MAPK signal pathway in the mutation spectrum (46.5%), but also found new potential driving mutation genes, including USP9X (15.5%), EIF1AX (2%), MACF1 (11.2%), ARID1A (9.9%), NF2 (4.2%), DOT1L (5.6%), and ASH1L (4.2%).^[6]^ USP9X and EIF1AX are related to the genes of AKT mTOR pathway and downstream effectors of the MAPK pathway.^[24]^ In addition to gene mutations, copy number aberrations are common in LGSOC. The loss of chromosome 9p, increase in chromosomes 8, 12, and 20, and the homozygous deletion of CDKN2A/B and MTAP are common. These changes in the copy number are related to the loss of P16 expression, which suggests the potential role of cell cycle inhibitors, such as cell cycle inhibitors targeting CDK4/6.^[16,25]^ Recently, Schütte et al^[26]^ found that the integration of genomic and transcriptome data is significantly better than a simple genomic analysis in identifying clinically relevant molecular changes. Shrestha et al^[27]^ used whole genome sequencing, RNA sequencing, and proteomics integration based on the mass spectrometry on 14 LGSOC cell lines to identify new biomarkers. Different somatic mutation patterns were found in the MEKi sensitive and MEKi resistant cell lines. KRAS mutations were only found in MEKi sensitive cell lines, while NRAS mutations were mainly found in MEKi resistant cell lines. In addition, the deletion of MTAP and CDKN2A/B genes is more frequent in cell lines than in tumor samples and may represent a key driving event in the absence of KRAS/BRAF/NRAS mutations. Because of the proximity of its chromosomes, it is often codeleted with CDKN2A/B. Whether the codeletion of MTAP and CDKN2A/B can be utilized through the synthetic lethal strategy is worth further exploration. Therefore, only by accurately describing the molecular changes of LGSOC, we can provide valuable guidance for clinical treatment. The tumor has cell heterogeneity and clonal evolution. The accuracy of the detection technology and the comprehensiveness of the information reflected by the detected objects, such as tumor tissue samples are key to accurately understand the molecular variation information of LGSOC. Further in-depth research on LGSOC genomics can provide valuable information for clinical treatment.

## 4. Rethinking the clinical treatment dilemma of LGSOC

The National Comprehensive Cancer Network (NCCN) guidelines recommend postoperative observation for patients with LGSOC in the IA and IB stages. Notably, patients in the IC stage can choose observation, chemotherapy, or endocrine therapy after operation; for LGSOC patients in stages II to IV, it is recommended that 6 cycles of carboplatin/paclitaxel adjuvant chemotherapy be followed by endocrine maintenance therapy (Grade 2 B) or endocrine therapy alone (Grade 2 B), which will last until disease progression or intolerable side effects occur, and bone loss and hyperlipidemia should be monitored during treatment.^[14]^ Most (70%) patients with advanced LGSOC experienced disease recurrence. After recurrence, a variety of treatment options are available, including secondary tumor cell reduction, chemotherapy, endocrine/hormone therapy, targeted therapy, and clinical trials. Simultaneously, if allowed, molecular sequencing is performed to obtain the somatic mutation spectrum to determine the best therapeutic target.^[28]^ Despite the continuous improvement in surgical skills, the increasing proportion of satisfactory tumor cell reduction operations, new chemotherapy drugs, targeted drugs, compatibility, and combinations of different schemes, the 5-year survival rate of patients with advanced LGSOC has not fundamentally improved in the past 20 years.^[7,29]^ Additionally, all clinical drug trials have been developed and tested on the basis of the HGSOC population, which cannot guide the treatment of LGSOC in a standardized way. This makes it necessary to reconsider the treatment dilemma of LGSOC.

## 5. Screening and early diagnosis

Fifty-eight percent of ovarian cancer had metastasis at the time of diagnosis, and the 5-year survival rate was only 30%. However, the 5-year survival rate for early stage ovarian cancer is 93%.^[30]^ This factor has spurred people to explore a screening tool to detect ovarian cancer as early as possible in asymptomatic women. Serum CA125 level and transvaginal ultrasound (TVU) are the most promising screening tools so far.^[31]^ The Prostate, Lung, Colorectal and Ovarian (PLCO) cancer screening trial is the largest prospective ovarian cancer screening trial in the United States, with more than 78,000 women participating. Using the biomarker CA125 combined with TVU for screening, a mortality benefit of screening was not identified,^[32]^ and it was found that screening might lead to overdiagnosis of benign ovarian tumors, which might have been detected in clinical or not detected at all, and if not detected by screening, the results would be similar. The larger UK Collaborative Trial of Ovarian Cancer Screening randomly divided 202,638 average-risk women into nonscreening, annual TVU or multimodal screening. The ovarian cancer risk algorithm was used to explain the serum CA125, and TVU was performed when necessary. The results showed fewer complications but again not a reduction in mortality from ovarian cancer screening.^[33]^ The possible delayed effects of screening need to be confirmed through long-term follow-up. During follow-up of the PLCO study, the results of the previous study were improved because the tissue subtypes of ovarian cancer were differentiated. Interestingly, compared with type II EOC, the survival rate of type I tumors at early diagnosis is improved, and can also be detected through screening.^[34]^ We think the probability of early detection of LGSOCs is much higher because these tumors take longer to develop from early to late stages. This was also supported by the high proportion of LGSOCs diagnosed in FIGO stage I/II. Recently, some scholars have discovered other protein biomarkers that may be used in combination with CA125 and other promising biomarkers, including autoantibodies,^[35]^ circulating tumor DNA,^[36,37]^ micro-RNAs,^[38,39]^ and DNA methylation.^[40,41]^ However, these biomarkers as screening tools need to be further developed, validated, and finally tested in randomized controlled trials.^[42]^

Screening methods that reflect the biology of low-grade serous ovarian cancer and better risk stratification are urgently needed. Determining which factors increase the risk of women suffering from ovarian cancer should be a priority of all screening work to accurately and effectively identify high-risk groups, which are expected to benefit from early monitoring and potential risk-reduction interventions, such as risk-reduction salpingo-oophorectomy (RRSO), which can be performed when women fully realize their reproductive aspirations.^[43]^ To this end, medical institutions are making efforts to determine the most relevant risk factors that affect cancer risk, such as genetic profiles, reproductive history, family background, lifestyle, and/or other clinical characteristics. The ongoing refinement of risk prediction models will undoubtedly improve targeted screening and diagnostic methods, as well as refine clinical decision-making for at-risk populations and guide their choice of risk-reduction methods, such as RRSO.^[43]^ Therefore, the screening of LGSOC is still elusive, and we still have a lot of work to do.

## 6. Surgery

Surgery is the cornerstone of treatment for LGSOC. Because of the important impact of LGSOC’s relative chemotherapy resistance and satisfactory cytoreductive surgery (CRS) on the prognosis, it highlights the importance of R0 resection.^[24]^ A meta-analysis of the AGO database of 145 LGSOC patients showed that the incidence of RD > 1 cm after CRS was 21.4%, 51.7% of patients reached R0, the 5-year survival rate of patients with RD > 1 cm was 32.0%, and that of patients with R0 was 85.0%.^[44]^ In addition, since the standard chemotherapy of NACT or platinum drugs and paclitaxel regimen has no significant improvement in the PFS or OS of LGSOC patients, the value of interval tumor CRS (NACT-IDS) after neoadjuvant chemotherapy in LGSOC is less than that in HGSOC. Until a more effective treatment is found, appropriate relaxation of the surgical indications for LGSOC patients who can tolerate surgery and performing surgery as much as possible to achieve R0 will result in greater survival benefits for LGSOC patients.

Regarding the significance of lymph node dissection in LGSOC, the NCCN guidelines in 2021 adopted the results of the ovarian tumor lymph node resection (LION) study, that is, for ovarian cancer patients with stage IIIb or above and negative lymph nodes, systematic lymph node resection is not allowed. However, most of the participants in the LION study were HGSOCs, and their conclusions cannot be unconditionally reasoned and applied to LGSOC. In principle, the 2 should be different in surgery. For LGSOC patients above stage IIIb, systematic lymphadenectomy as part of the initial comprehensive staging surgery should not be ignored. Gockley et al^[45]^ conducted a retrospective study on a large sample size of patients with advanced serous ovarian cancer from 2003 to 2011 through the National Cancer Database and analyzed the value of lymph node dissection in 755 patients with advanced LGSOC and found that the median OS of patients receiving lymphadenectomy was significantly longer than that of patients without lymphadenectomy (106.5 and 58.0 months, respectively). It can be seen that systematic lymphadenectomy is of great significance for LGSOC patients. When medical technology is allowed and patients are generally in good condition, systematic lymphadenectomy should be routinely performed as part of the comprehensive staging surgery.

Regarding the value of secondary cytoreductive surgery (SCRS), the general trend was that SCRS may improve the prognosis of LGSOC patients with R0; however, RD size was a poor prognostic factor if SCRS did not reach R0.^[46]^ For patients with their third, fourth, or fifth recurrence, individualized surgery may be considered. A review of previous studies suggests that repeat surgery is feasible and may be beneficial.^[47]^ Therefore, in the absence of more effective nonsurgical treatments for LGSOC, tumor reduction should be actively implemented.

## 7. Chemotherapy

The retrospective study of the AGO database showed that the initial chemotherapy resistance rate of LGSOC was 4% to 25%,^[24]^ and it showed a relative chemical resistance to neoadjuvant chemotherapy,^[48]^ adjuvant chemotherapy,^[44]^ and recurrent chemotherapy.^[49]^ Because of the low responsiveness of LGSOC to neoadjuvant chemotherapy, most experts and scholars do not recommend neoadjuvant chemotherapy for LGSOC; however, some LGSOC patients do have an extensive tumor load, and the difference in the overall survival between patients receiving PDS and IDS seems to be more related to the degree of disease, patient selection, and poorer tumor biology. This is not only due to differences in surgical timing.^[48]^ In addition, LGSOC must be definitively diagnosed by biopsy prior to neoadjuvant chemotherapy and not just by cytology. In the case of recurrence, LGSOC has a lower response rate to chemotherapy (5%).^[49]^ However, the disease stabilization rate can be as high as 60%, which is attributed to the inert biological characteristics of the tumor or the unclear effect of chemotherapy. Further clinical trials are needed to confirm the clinical benefits of chemotherapy. In view of the lack of strong evidence for alternative chemotherapy regimens, most chemotherapy regimens chosen for treatment are similar to HGSOC.^[50]^ In vitro experimental studies have shown that LGSOC has strong resistance to paclitaxel, carboplatin, cisplatin, cyclophosphamide, and gemcitabine and weak resistance to etoposide, adriamycin, and topotecan.^[49]^ Rose et al^[51]^ evaluated 21 LGSOC patients with PLD application and found that 3 patients (14.3%) achieved CR after the application of PLD, 67% of the patients had stable disease, and the response rate to PLD was independent of platinum sensitivity. PLD showed good application prospects. In addition, some researchers^[52,53]^ identified a new LGSOC-related drug resistance gene, CLU. This gene encodes a secreted protein that prevents paclitaxel-induced apoptosis of ovarian cancer cell lines. Therefore, the inhibition of CLU expression and reversal of paclitaxel resistance have the potential to increase the sensitivity of LGSOC to paclitaxel-based adjuvant therapy, and combination therapy, including CLU inhibitors may increase the clinical response of LGSOC patients to standard chemotherapy.

## 8. Endocrine therapy

Approximately 95% of LGSOC patients have tumor ER expression and 50% have PR expression, which represents a potential therapeutic target.^[24]^ Gershenson et al^[54]^ found that the PFS of stage II to IV LGSOC patients receiving hormone maintenance therapy after the initial treatment was significantly longer than that of patients in the observation group (26.4 months vs 64.9 months). A further subgroup analysis showed that high-risk groups with residual lesions after the end of treatment had greater clinical benefits from hormone maintenance therapy. To investigate the need for chemotherapy in LGSOC patients receiving hormone maintenance therapy, a phase II clinical trial (NRG-GY-019) randomized newly diagnosed stage II to IV LGSOC patients after primary tumor reduction to either trozole maintenance after standard adjuvant chemotherapy or letrozole alone. This study had a noninferiority design. Launched in June 2019, it may reveal whether patients receiving hormone maintenance therapy also require chemotherapy.^[24]^ In relapsed LGSOC, the response rate to hormone therapy is approximately 9% to 14%, and the disease stabilization rate is 50% to 60%.^[54,55]^ Although hormone therapy has shown some anticancer activity in relapsed LGSOC, the overall response rate remains low. Some scholars have found that patients with ER/PR expression have a good prognosis^[56]^; however, there is still no exact evidence that ER/PR can predict the clinical benefit of hormone therapy in recurrent LGSOC, and biomarkers for predicting the clinical benefit of hormone therapy still need to be further explored.^[57]^ In addition, up to 50% of ERα-positive LGSOC patients are resistant to hormone therapy, and it has been found that mutations in the ESR1 gene encoding ERα can lead to resistance to endocrine therapy, especially aromatase inhibitors (AI).^[58]^ Whether the ESR1 mutation status can be considered a predictive biomarker or a biomarker related to clinical trials is worthy of further exploration. Other researchers have found that MAPK activation in ovarian cancer model tumor cells can drive hormone therapy resistance through ERα.^[56]^ Therefore, MEKi and endocrine drugs may have a synergistic effect, and future clinical trials of MERi combined with AI are needed to further verify this.^[59]^ In addition, a clinical trial of AI in combination with a cyclin-dependent kinase inhibitor (NCT03673124) is underway to evaluate the efficacy of letrozole and the CDK4/6 inhibitor, ribociclib, in relapsed LGSOC with measurable lesions.

## 9. Exploration of new therapeutic strategies

Given the prevalence of MAPK pathway alterations in patients with LGSOC, several clinical trials have explored the efficacy of MEKi in LGSOC. The first MEKi prospective clinical trial in LGSOC was a single-arm phase II trial of oral smetinib in patients with relapse.^[60]^ This study showed that the objective response rate was 15% and the median PFS of 11 months was 15%. Since only 34 of 52 (65%) patients had sufficient tumor DNA to be used for a BRAF/KRAS mutation analysis, there was no exact evidence to show that there was an association between the mutation status and treatment response. Subsequently, 2 phase III clinical studies (MILO study/NCT02101788) attempted to compare MEKi with the standard treatment for patients with recurrent LGSOC. The MILO Study was conducted in 2013. A total of 341 patients from 17 countries were enrolled. The patients were randomly assigned to receive binimetinib (340 mg bid) or chemotherapy selected by the doctors. In the MEKi group, the progression-free survival of patients with KRAS mutation was significantly longer than that of patients with wild type (17.7 months vs 10.8 months, *P* = .006); In the chemotherapy group, the median PFS of KRAS mutation patients was 14.6 months, and that of wild-type patients was 11.5 months (*P* = .502); In all patient populations, the binimetinib group did not show PFS benefits. A factorial analysis showed that KRAS mutations could predict the clinical benefits of binimetinib. Patients with KRAS mutations have a progression-free survival benefit of nearly 7 months.^[61]^ In addition, some scholars have reported that the benefits of the chemotherapy group in this study exceeded expectations. Another phase III clinical trial (NCT02101788) compared MEKi (trimetinib) with standard treatments (letrozole, polyethylene glycol liposome doxorubicin, paclitaxel, tamoxifen, or topotecan) for patients with recurrent LGSOC. Detailed results were recently published by *The Lancet*.^[62]^ The median PFS in the trimetinib group was 13.0 months, while that in the standard treatment group was 7.2 months (hazard ratio [HR] = 0.48; 95% CI: 0.36–0.64; unilateral *P* < .0001). Compared to the current standard treatment, trimetinib can reduce the risk of disease progression or death in patients with recurrent or persistent LGSOC by 52%. Trimetinib is the most favorable choice after the first recurrence of first-line chemotherapy and sequential AI treatment, and may become a potential new standard treatment. Similar to the MILO study, this study also found that patients with KRAS, BRAF, or NRAS mutations had significantly longer median PFS (13.2 months vs 7.3 months) and significantly higher ORR (50.0% vs 8.3%) than patients with wild-type KRAS, BRAF, or NRAS. However, this study still believes that MEKi should be considered as the choice for all patients with recurrent LGSOC, regardless of the mutation status, based on the lack of clear information about the relationship between mutation status and treatment response and the best predictive biomarker panel.

MEKi affects the MAPK pathway and inhibits cell proliferation mainly through its effect on the MEK protein; however, it is often prone to drug resistance. Signaling pathways form regulatory “circuits” through positive and negative feedback, leading to acquired or adaptive resistance. Therefore, it may be effective to more accurately describe MAPK signals, better understand the pathway feedback loop, and ultimately use this information in the rational design of combination therapies to target multiple factors in the pathway, or simultaneously target other pathways.^[57]^ A phase I FRAME trial led by the Royal Marsden Hospital and the British Cancer Research Institute (ICR) research team aimed to solve the problem of MEKi drug resistance. A total of 25 LGSOC patients were tested for VS-6766 (a RAF/MEK dual inhibitor) in combination with the FAK inhibitor defactinib. The results of this study were published in ESMO in 2021. Approximately half (46%) of the patients had significantly reduced tumors, and the effect of patients with KRAS gene mutations was more optimistic, with 64% of patients having smaller tumors. A phase II trial is currently recruiting patients to further test the effectiveness of this drug combination. At the same time, another phase II clinical trial (SAR24509) compared the effect of MEKi (pimasertib) combined with a PI3K/mTOR inhibitor in patients with inoperable recurrent LGSOC. The control group received a placebo. This clinical trial has been terminated and the results were promising. In addition, antiangiogenic drugs, such as bevacizumab monotherapy or combination therapy, also show good application prospects, with a single drug response rate of 48%^[63,64]^; however, the efficacy needs to be further verified in clinical practice.

## 10. Conclusions

Surgery is the cornerstone of LGSOC treatment and maximum effort must be made to achieve R0 removal. Although LGSOC is not sensitive to chemotherapy, postoperative platinum-based combination chemotherapy remains the first-line treatment option for LGSOC. Additional clinical trials are needed to confirm the clinical benefits of chemotherapy and explore new chemotherapy protocols. Hormone and targeted therapies may also play important roles. Some patients, particularly those with residual lesions after treatment, may benefit from hormone maintenance therapy after chemotherapy. Targeted therapies, such as MEKi, show good application prospects and are expected to change the treatment pattern of LGSOC. Continuing to further study the genomics of LGSOC, identify its specific gene changes, and combine traditional treatment methods with precision targeted therapy based on second-generation sequencing may be the direction for LGSOC to overcome the treatment bottleneck. In the future, the treatment of LGSOC will increasingly use individualized “precision medicine according to different tumor characteristics of each patient, and patients will benefit significantly from the most appropriate individualized “precision therapy.”^[65]^

## Acknowledgements

Thank all participants for their kindly help during the formation and finalization processes of the manuscript.

## Author contributions

**Conceptualization:** Bei Zhang.

**Data curation:** Jing-Bo Zhang.

**Funding acquisition:** Bei Zhang.

**Methodology:** Yan-Yu Li.

**Resources:** Xin-Hui Yang.

**Supervision:** Bei Zhang.

**Validation:** Jing-Bo Zhang.

**Visualization:** Xin-Hui Yang, Bei Zhang.

**Writing—original draft:** Qing Wang.

**Writing—review & editing:** Sheng-Han Cao.

## References

[1] Kurman RJ Shih Ie-M. The dualistic model of ovarian carcinogenesis: revisited, revised, and expanded. Am J Pathol. 2016;186:733–47. 27012190 10.1016/j.ajpath.2015.11.011 PMC5808151

[2] Singer G Kurman RJ Chang HW. Diverse tumorigenic pathways in ovarian serous carcinoma. Am J Pathol. 2002;160:1223–8. 11943707 10.1016/s0002-9440(10)62549-7 PMC1867233

[3] Shih IeM Kurman RJ. Ovarian tumorigenesis: a proposed model based on morphological and molecular genetic analysis. Am J Pathol. 2004;164:1511–8. 15111296 10.1016/s0002-9440(10)63708-x PMC1615664

[4] Gershenson DM. Low-grade serous carcinoma of the ovary or peritoneum. Ann Oncol. 2016;27(Suppl 1):i45–9. 27141071 10.1093/annonc/mdw085

[5] Kaldawy A Segev Y Lavie O. Low-grade serous ovarian cancer: a review. Gynecol Oncol. 2016;143:433–8. 27581327 10.1016/j.ygyno.2016.08.320

[6] Cheasley D Nigam A Zethoven M. Genomic analysis of low-grade serous ovarian carcinoma to identify key drivers and therapeutic vulnerabilities. J Pathol. 2021;253:41–54. 32901952 10.1002/path.5545

[7] Siegel RL Miller KD Fuchs HE. Cancer statistics, 2022. CA Cancer J Clin. 2022;72:7–33. 35020204 10.3322/caac.21708

[8] Qiu C Lu N Wang X. Gene expression profiles of ovarian low-grade serous carcinoma resemble those of fallopian tube epithelium. Gynecol Oncol. 2017;147:634–41. 28965696 10.1016/j.ygyno.2017.09.029

[9] Ahmed N Abubaker K Findlay J. Cancerous ovarian stem cells: obscure targets for therapy but relevant to chemoresistance. J Cell Biochem. 2013;114:21–34. 22887554 10.1002/jcb.24317

[10] Marquez RT Baggerly KA Patterson AP. Patterns of gene expression in different histotypes of epithelial ovarian cancer correlate with those in normal fallopian tube, endometrium, and colon. Clin Cancer Res. 2005;11:6116–26. 16144910 10.1158/1078-0432.CCR-04-2509

[11] Vang R Shih IeM Kurman RJ. Fallopian tube precursors of ovarian low- and high-grade serous neoplasms. Histopathology. 2013;62:44–58. 23240669 10.1111/his.12046

[12] Kurman RJ Vang R Junge J. Papillary tubal hyperplasia: the putative precursor of ovarian atypical proliferative (borderline) serous tumors, noninvasive implants, and endosalpingiosis. Am J Surg Pathol. 2011;35:1605–14. 21997682 10.1097/PAS.0b013e318229449f PMC3193599

[13] Wang Y Hong S Mu J. Tubal origin of “ovarian” low-grade serous carcinoma: a gene expression profile study. J Oncol. 2019;2019:8659754. 30949203 10.1155/2019/8659754 PMC6425354

[14] Babaier A Mal H Alselwi W. Low-grade serous carcinoma of the ovary: the current status. Diagnostics (Basel). 2022;12:458. 35204549 10.3390/diagnostics12020458 PMC8871133

[15] Jones S Wang TL Kurman RJ. Low-grade serous carcinomas of the ovary contain very few point mutations. J Pathol. 2012;226:413–20. 22102435 10.1002/path.3967 PMC3503448

[16] Hunter SM Anglesio MS Ryland GL.; Australian Ovarian Cancer Study Group. Molecular profiling of low grade serous ovarian tumours identifies novel candidate driver genes. Oncotarget. 2015;6:37663–77. 26506417 10.18632/oncotarget.5438 PMC4741956

[17] Etemadmoghadam D Azar WJ Lei Y.; Australian Ovarian Cancer Study Group. EIF1AX and NRAS mutations co-occur and cooperate in low-grade serous ovarian carcinomas. Cancer Res. 2017;77:4268–78. 28646021 10.1158/0008-5472.CAN-16-2224

[18] Romero I Sun CC Wong KK. Low-grade serous carcinoma: new concepts and emerging therapies. Gynecol Oncol. 2013;130:660–6. 23707670 10.1016/j.ygyno.2013.05.021

[19] Singer G Oldt R 3rd Cohen Y. Mutations in BRAF and KRAS characterize the development of low-grade ovarian serous carcinoma. J Natl Cancer Inst. 2003;95:484–6. 12644542 10.1093/jnci/95.6.484

[20] Grisham RN Iyer G Garg K. BRAF mutation is associated with early stage disease and improved outcome in patients with low-grade serous ovarian cancer. Cancer. 2013;119:548–54. 22930283 10.1002/cncr.27782 PMC3961140

[21] Prat J D’angelo E Espinosa I. Ovarian carcinomas: at least five different diseases with distinct histological features and molecular genetics. Hum Pathol. 2018;80:11–27. 29944973 10.1016/j.humpath.2018.06.018

[22] Emmanuel C Chiew YE George J.; Australian Ovarian Cancer Study (AOCS). Genomic classification of serous ovarian cancer with adjacent borderline differentiates RAS pathway and TP53-mutant tumors and identifies NRAS as an oncogenic driver. Clin Cancer Res. 2014;20:6618–30. 25316818 10.1158/1078-0432.CCR-14-1292

[23] Manning-Geist B Gordhandas S Liu YL. MAPK pathway genetic alterations are associated with prolonged overall survival in low-grade serous ovarian carcinoma. Clin Cancer Res. 2022;28:4456–65. 35443055 10.1158/1078-0432.CCR-21-4183 PMC9582036

[24] Slomovitz B Gourley C Carey MS. Low-grade serous ovarian cancer: state of the science. Gynecol Oncol. 2020;156:715–25. 31969252 10.1016/j.ygyno.2019.12.033

[25] Van Nieuwenhuysen E Busschaert P Laenen A. Loss of 1p36.33 frequent in low-grade serous ovarian cancer. Neoplasia. 2019;21:582–90. 31054497 10.1016/j.neo.2019.03.014 PMC6500912

[26] Schütte M Risch T Abdavi-Azar N. Molecular dissection of colorectal cancer in pre-clinical models identifies biomarkers predicting sensitivity to EGFR inhibitors. Nat Commun. 2017;8:14262. 28186126 10.1038/ncomms14262 PMC5309787

[27] Shrestha R Llaurado Fernandez M Dawson A. Multiomics characterization of low-grade serous ovarian carcinoma identifies potential biomarkers of MEK inhibitor sensitivity and therapeutic vulnerability. Cancer Res. 2021;81:1681–94. 33441310 10.1158/0008-5472.CAN-20-2222

[28] Voutsadakis IA. Low-grade serous ovarian carcinoma: an evolution toward targeted therapy. Int J Gynecol Cancer. 2020;30:1619–26. 31780569 10.1136/ijgc-2019-000832

[29] Slomski A. Screening women for ovarian cancer still does more harm than good. JAMA. 2012;307:2474–5. 22797429 10.1001/jama.2012.5646

[30] Siegel RL Miller KD Fuchs HE. Cancer statistics, 2021. CA Cancer J Clin. 2021;71:7–33. 33433946 10.3322/caac.21654

[31] Hurwitz LM Pinsky PF Trabert B. General population screening for ovarian cancer. Lancet. 2021;397:2128–30. 33991478 10.1016/S0140-6736(21)01061-8

[32] Buys SS Partridge E Black A.; PLCO Project Team. Effect of screening on ovarian cancer mortality: the Prostate, Lung, Colorectal and Ovarian (PLCO) cancer screening randomized controlled trial. JAMA. 2011;305:2295–303. 21642681 10.1001/jama.2011.766

[33] Menon U Gentry-Maharaj A Burnell M. Ovarian cancer population screening and mortality after long-term follow-up in the UK Collaborative Trial of Ovarian Cancer Screening (UKCTOCS): a randomised controlled trial. Lancet. 2021;397:2182–93. 33991479 10.1016/S0140-6736(21)00731-5 PMC8192829

[34] Temkin SM Miller EA Samimi G. Outcomes from ovarian cancer screening in the PLCO trial: histologic heterogeneity impacts detection, overdiagnosis and survival. Eur J Cancer. 2017;87:182–8. 29156299 10.1016/j.ejca.2017.10.015

[35] Shang Y Zeng Y Zeng Y. Integrated microfluidic lectin barcode platform for high-performance focused glycomic profiling. Sci Rep. 2016;6:20297. 26831207 10.1038/srep20297 PMC4735825

[36] Corcoran RB Chabner BA. Application of cell-free DNA analysis to cancer treatment. N Engl J Med. 2018;379:1754–65. 30380390 10.1056/NEJMra1706174

[37] Wan JCM Massie C Garcia-Corbacho J. Liquid biopsies come of age: towards implementation of circulating tumour DNA. Nat Rev Cancer. 2017;17:223–38. 28233803 10.1038/nrc.2017.7

[38] Elias KM Fendler W Stawiski K. Diagnostic potential for a serum miRNA neural network for detection of ovarian cancer. Elife. 2017;6:e28932. 29087294 10.7554/eLife.28932 PMC5679755

[39] Kandimalla R Wang W Yu F. OCaMIR – a noninvasive, diagnostic signature for early-stage ovarian cancer: a multi-cohort retrospective and prospective study. Clin Cancer Res. 2021;27:4277–86. 34035068 10.1158/1078-0432.CCR-21-0267 PMC10327469

[40] Dammann RH Kirsch S Schagdarsurengin U. Frequent aberrant methylation of the imprinted IGF2/H19 locus and LINE1 hypomethylation in ovarian carcinoma. Int J Oncol. 2010;36:171–9. 19956846

[41] Pisanic TR 2nd Asaka S Lin SF. Long interspersed nuclear element 1 retrotransposons become deregulated during the development of ovarian cancer precursor lesions. Am J Pathol. 2019;189:513–20. 30553834 10.1016/j.ajpath.2018.11.005 PMC6412403

[42] Nebgen DR Lu KH Bast RC Jr. Novel approaches to ovarian cancer screening. Curr Oncol Rep. 2019;21:75. 31346778 10.1007/s11912-019-0816-0 PMC6662655

[43] Liberto JM Chen SY Shih IM. Current and emerging methods for ovarian cancer screening and diagnostics: a comprehensive review. Cancers (Basel). 2022;14:2885. 35740550 10.3390/cancers14122885 PMC9221480

[44] Grabowski JP Harter P Heitz F. Operability and chemotherapy responsiveness in advanced low-grade serous ovarian cancer. An analysis of the AGO Study Group metadatabase. Gynecol Oncol. 2016;140:457–62. 26807488 10.1016/j.ygyno.2016.01.022

[45] Gockley A Melamed A Bregar AJ. Outcomes of women with high-grade and low-grade advanced-stage serous epithelial ovarian cancer. Obstet Gynecol. 2017;129:439–47. 28178043 10.1097/AOG.0000000000001867 PMC5328143

[46] Crane EK Sun CC Ramirez PT. The role of secondary cytoreduction in low-grade serous ovarian cancer or peritoneal cancer. Gynecol Oncol. 2015;136:25–9. 25448453 10.1016/j.ygyno.2014.11.005 PMC4815920

[47] Schmeler KM Sun CC Bodurka DC. Neoadjuvant chemotherapy for low-grade serous carcinoma of the ovary or peritoneum. Gynecol Oncol. 2008;108:510–4. 18155273 10.1016/j.ygyno.2007.11.013

[48] Cobb LP Sun CC Iyer R. The role of neoadjuvant chemotherapy in the management of low-grade serous carcinoma of the ovary and peritoneum: Further evidence of relative chemoresistance. Gynecol Oncol. 2020;158:653–8. 32709538 10.1016/j.ygyno.2020.06.498

[49] Gershenson DM Sun CC Bodurka D. Recurrent low-grade serous ovarian carcinoma is relatively chemoresistant. Gynecol Oncol. 2009;114:48–52. 19361839 10.1016/j.ygyno.2009.03.001

[50] May T Shoni M Crum CP. Low-grade and high-grade serous Mullerian carcinoma: review and analysis of publicly available gene expression profiles. Gynecol Oncol. 2013;128:488–92. 23253401 10.1016/j.ygyno.2012.12.009

[51] Rose PG Radeva M Michener CM. Efficacy of pegylated liposomal doxorubicin in low-grade serous ovarian carcinoma. Int J Gynecol Cancer. 2017;27:907–11. 28498259 10.1097/IGC.0000000000000977

[52] Park DC Yeo SG Wilson MR. Clusterin interacts with Paclitaxel and confer Paclitaxel resistance in ovarian cancer. Neoplasia. 2008;10:964–72. 18714397 10.1593/neo.08604 PMC2517641

[53] Koltai T. Clusterin: a key player in cancer chemoresistance and its inhibition. Onco Targets Ther. 2014;7:447–56. 24672247 10.2147/OTT.S58622 PMC3964162

[54] Gershenson DM Bodurka DC Coleman RL. Hormonal maintenance therapy for women with low-grade serous cancer of the ovary or peritoneum. J Clin Oncol. 2017;35:1103–11. 28221866 10.1200/JCO.2016.71.0632 PMC5455356

[55] Tang M O’connell RL Amant F. PARAGON: a phase II study of anastrozole in patients with estrogen receptor-positive recurrent/metastatic low-grade ovarian cancers and serous borderline ovarian tumors. Gynecol Oncol. 2019;154:531–8. 31227223 10.1016/j.ygyno.2019.06.011

[56] Llaurado Fernandez M Dawson A Kim H. Hormone receptor expression and outcomes in low-grade serous ovarian carcinoma. Gynecol Oncol. 2020;157:12–20. 31954537 10.1016/j.ygyno.2019.11.029

[57] Hou JY Rodriguez-Gabin A Samaraweera L. Exploiting MEK inhibitor-mediated activation of ERα for therapeutic intervention in ER-positive ovarian carcinoma. PLoS One. 2013;8:e54103. 23390495 10.1371/journal.pone.0054103 PMC3563537

[58] Fader AN Bergstrom J Jernigan A. Primary cytoreductive surgery and adjuvant hormonal monotherapy in women with advanced low-grade serous ovarian carcinoma: reducing overtreatment without compromising survival? Gynecol Oncol. 2017;147:85–91. 28768570 10.1016/j.ygyno.2017.07.127

[59] Hew KE Miller PC El-Ashry D. MAPK activation predicts poor outcome and the MEK inhibitor, selumetinib, reverses antiestrogen resistance in ER-positive high-grade serous ovarian cancer. Clin Cancer Res. 2016;22:935–47. 26482043 10.1158/1078-0432.CCR-15-0534 PMC4755805

[60] Farley J Brady WE Vathipadiekal V. Selumetinib in women with recurrent low-grade serous carcinoma of the ovary or peritoneum: an open-label, single-arm, phase 2 study. Lancet Oncol. 2013;14:134–40. 23261356 10.1016/S1470-2045(12)70572-7 PMC3627419

[61] Monk BJ Grisham RN Banerjee S. MILO/ENGOT-ov11: binimetinib versus physician’s choice chemotherapy in recurrent or persistent low-grade serous carcinomas of the ovary, fallopian tube, or primary peritoneum. J Clin Oncol. 2020;38:3753–62. 32822286 10.1200/JCO.20.01164 PMC7655017

[62] Gershenson DM Miller A Brady WE. Trametinib versus standard of care in patients with recurrent low-grade serous ovarian cancer (GOG 281/LOGS): an international, randomised, open-label, multicentre, phase 2/3 trial. Lancet. 2022;399:541–53. 35123694 10.1016/S0140-6736(21)02175-9 PMC8819271

[63] Dalton HJ Fleming ND Sun CC. Activity of bevacizumab-containing regimens in recurrent low-grade serous ovarian or peritoneal cancer: a single institution experience. Gynecol Oncol. 2017;145:37–40. 28139261 10.1016/j.ygyno.2017.01.027 PMC5884069

[64] Grisham RN Iyer G Sala E. Bevacizumab shows activity in patients with low-grade serous ovarian and primary peritoneal cancer. Int J Gynecol Cancer. 2014;24:1010–4. 24978709 10.1097/IGC.0000000000000190 PMC4401424

[65] Rodriguez-Freixinos V Lheureux S Mandilaras V. Impact of somatic molecular profiling on clinical trial outcomes in rare epithelial gynecologic cancer patients. Gynecol Oncol. 2019;153:304–11. 30792002 10.1016/j.ygyno.2019.02.005

